# Training Load, Immune Status, and Clinical Outcomes in Young Athletes: A Controlled, Prospective, Longitudinal Study

**DOI:** 10.3389/fphys.2018.00120

**Published:** 2018-03-23

**Authors:** Katharina Blume, Nina Körber, Dieter Hoffmann, Bernd Wolfarth

**Affiliations:** ^1^Department of Sports Medicine, Humboldt-University, Charité University Medicine, Berlin, Germany; ^2^Institute of Virology, Technische Universität München, Helmholtz Zentrum München, Munich, Germany

**Keywords:** EBV, URTI, athlete, training load, infection, susceptibility, stress, immune system

## Abstract

**Introduction:** Beside positive effects on athlete's health, competitive sport can be linked with an increased risk of illness and injury. Because of high relative increases in training, additional physical and psychological strains, and an earlier specialization and professionalization, adolescent athletes needs an increased attention. Training can alter the immune system by inducing a temporary immunosuppression, finally developing infection symptoms. Previous studies identified Epstein Barr Virus (EBV) as potential indicator for the immune status. In addition to the identification of triggering risk factors for recurrent infections, the aim was to determine the interaction between training load, stress sense, immunological parameters, and clinical symptoms.

**Methods:** A controlled, prospective, longitudinal study on young athletes (*n* = 274, mean age: 13.8 ± 1.5 yrs) was conducted between 2010 and 2014. Also 285 controls (students, who did not perform competitive sports, mean age: 14.5 ± 1.9 yrs) were recruited. Athletes were examined 3 times each year to determine the effects of stress factors (training load: training hours per week [Th/w]) on selected outcome parameters (clinical [susceptibility to infection, WURSS-21: 21-item *Wisconsin Upper Respiratory Symptom Survey*], immunological, psychological end points). As part of each visit, EBV serostatus and EBV-specific IgG tiers were studied longitudinally as potential immune markers.

**Results:** Athletes (A) trained 14.9 ± 5.6 h weekly. Controls (C) showed no lower stress levels compared to athletes (*p* = 0.387). Twelve percent of athletes reported recurrent infections (C: 8.5%, *p* = 0.153), the presence of an upper respiratory tract infection (URTI) was achieved in 30.7%. EBV seroprevalence of athletes was 60.3% (C: 56.6%, *p* = 0.339). Mean EBV-specific IgG titer of athletes was 166 ± 115 U/ml (C: 137 ± 112 U/ml, *p* = 0.030). With increasing Th/w, higher stress levels were observed (*p* < 0.001). Analyzes of WURSS-21 data revealed no relationship to training load (*p* = 0.323). Also, training load had no relation to EBV serostatus (*p* = 0.057) or the level of EBV-specific IgG titers (*p* = 0.364).

**Discussion:** Young elite athletes showed no increased sense of stress, no higher prevalence of recurrent infections, and no different EBV-specific serological parameters compared to controls. Also, no direct relationship between training loads, clinical complaints, and EBV-specific immune responses was found. With increasing training loads athletes felt more stressed, but significant associations to EBV-specific serological parameters were absent. In summary, EBV serostatus and EBV-specific IgG titers do not allow risk stratification for impaired health. Further investigations are needed to identify additional risk factors and immune markers, with the aim to avoid inappropriate strains by early detection and following intervention.

## Introduction

Competitive sport is associated with various physical and psychological strains (Sabato et al., 2016; Schwellnus et al., 2016a,b). Beside positive effects on athlete's health and well-being, including cardiovascular and muscular fitness, bone health, weight control, psychosocial outcomes, cognitive and brain health, and reduced morbidity and premature death (Wartburton and Bredin, 2016, 2017; Chieffi et al., 2017), acute prolonged exercise can be linked with an increased risk of illness and injury (Armstrong and Mc Manus, 2011; Hastmann-Walsh and Caine, 2015). Success requires numerous years of training, starting already in adolescence, with presumed health and less lack of practice to achieve sports specific skills and necessary overall capacity (Mårtensson et al., 2014). The trend of recent years shows an increased duration, intensity, and difficulty of training, a high-frequency participation in sports events, and an earlier specialization and professionalization (Mountjoy et al., 2008; Caine, 2010; Armstrong and Mc Manus, 2011). Such conditions can negatively affect the risk of physical and psychological illness and injury (Armstrong and Mc Manus, 2011; Sabato et al., 2016). Therefore, to ensure resilience in young athletes, age-based additional endo- and exogenous risk factors, which can negatively influence the ability to withstand stress, should be known and considered: e.g., physical development, high training load (DiFiori and Mandelbaum, 1996; Dennis et al., 2005; Dun et al., 2005; Loud et al., 2005; Fleisig et al., 2011; Hjelm et al., 2012), early specialization (Barynina and Vaitsekhovskii, 1992; Bompa, 1995; Jayanthi et al., 2013), performance capacity, previous illnesses, environmental factors, and negative stressors such as school, parental conflicts, pressure to perform, and competition failure (Cohn, 1990; Scanlan et al., 1991; Puffer and McShane, 1992; Gould et al., 1993; Puente-Diaz and Anshel, 2005). Ignoring the multifactorial interactions of various triggers can lead to a diminished immune competence affecting health, training and ability for regeneration (Borresen and Lambert, 2009; Dhabhar, 2014). Light clinical symptoms over one episode usually result in short training breaks. In contrast, recurrent infections, mild or severe, can cause frequent interruptions, lack or stagnation of performance, retirement from competitive sports, furthermore, influencing long-term everyday life (Maffulli et al., 2010; Whittaker et al., 2015).

Upper respiratory tract infections (URTI) belong to the most common diseases in athletes (Gleeson and Pyne, 2016; Schwellnus et al., 2016a). Beside musculoskeletal injuries (Schwellnus et al., 2016b), they are the main cause responsible for training failures, suboptimal performances, and competition cancellations (Fricker, 1997; Alonso et al., 2010; Engebretsen et al., 2010, 2013; Mountjoy et al., 2010; Schwellnus et al., 2011, 2016a; Soligard et al., 2014). During the Winter Olympics 2014 in Sotschi, 8.9% of athletes had an illness, 64% of them an URTI as reason (Soligard et al., 2014). While the prevalence of URTI is comparable to the general population, an increased rate of susceptibility was found in athletes (Gleeson et al., 1995; Fricker et al., 2000). Studies have shown that moderate exercise reduces the incidence of infections compared to physical inactivity (Matthews et al., 2002). In contrast, high-intensity or rather extensive training loads are associated with an increased susceptibility to infections due to a diminished transient immune competence (Peters and Bateman, 1983; Fricker et al., 1999; Gleeson et al., 2000; Spence et al., 2007). The duration of this mentioned immunosuppression takes several hours, called as >open window< (Walsh and Oliver, 2016). Training, particularly high intensities, and marked load increases, can induce this temporary immunosuppression causing recurrent infections (Gleeson et al., 2000; Konig et al., 2000; Tiollier et al., 2005; Tsai et al., 2011; Walsh et al., 2011a; Hellard et al., 2015). Nevertheless, other potentially triggers and promotive risk factors must also be considered, such as previous illnesses (e.g., bronchial asthma; Reid et al., 2004; Spence et al., 2007; Cox et al., 2008), female gender (Himmelstein et al., 1998; Konig et al., 2000; He et al., 2014), age (Monto, 2002), genetic predispositions (Cox et al., 2010; Zehsaz et al., 2014), low IgA secretion rates (Gleeson et al., 1995, 1999; Putlur et al., 2004; Fahlman and Engels, 2005; Nieman et al., 2006), air travel (Svendsen et al., 2016), cold (Walsh and Oliver, 2016), heat (Walsh and Oliver, 2016), hypoxia (Walsh and Oliver, 2016), stress (Novas et al., 2002; Putlur et al., 2004; Main et al., 2010), lack of sleep (Cohen et al., 2009; Main et al., 2010), malnutrition (Zapico et al., 2007; Walsh et al., 2011b; Calder et al., 2014), and weight loss (Umeda et al., 2004; Shimizu et al., 2011). So far, it remains still controversial which factors, degree (e.g., duration, intensity, frequency), and attendant circumstances must be present for affecting immune system and finally developing clinical complaints (Fricker et al., 2000; Konig et al., 2000).

In addition to the question which factors lead to an impaired health and performance, it is necessary to quantify immunological parameters that potentially indicate a risk early. The clinical relevance of such immunological changes remains controversial. So far, no direct association between immune responses and increased infection rates could be clearly demonstrated (Reid et al., 2004; Fricker et al., 2005; Helenius et al., 2005; Tiollier et al., 2005; Cox et al., 2008). Consistently, physical stress activates the immune system more or less, depending on amount, intensity and frequency, resulting in weakness or stabilization. An association between low saliva IgA concentrations or rather reduced secretion rates and URTI symptoms has been demonstrated (Gleeson et al., 1995, 1999; Putlur et al., 2004; Fahlman and Engels, 2005; Nieman et al., 2006). Nevertheless, the results are inconsistent and the quantitation requires complex and time-consuming demands (Gleeson, 2000; Walsh et al., 2011a). A limitation that makes it difficult to use in practice. Therefore, simple, non-invasive and feasible tools should be developed including parameters that influence and reflect the immunological aspect of the individual capacity and resilience.

Diminished performance and fatigue with concurrent unspecific flu-like symptoms are often associated with an Epstein Barr Virus (EBV) infection in competitive athletes (Gleeson et al., 2002; Balfour et al., 2015). This herpes virus persists lifelong in the organism and is controlled by the adaptive immune system. The detection of antibodies to specific EBV antigens by immunoblot allows to determine the infection stage (De Paschale and Clerici, 2012). In contrast to other viruses (e.g., varicella-zoster virus, cytomegalovirus), replication of EBV occurs frequently by reactivation, intermittently or even continuously. Thus, in addition to EBV-specific antibodies, the viral genome is also well accessible for polymerase chain reaction (PCR, viral load), allowing the detection of a systemically increased EBV activity in whole blood or saliva (Yamauchi et al., 2011). The lifelong persistence in the organism after primary infection and a prevalence of more than 90% in adults, and between 55 and 80% in adolescent (Karrer and Nadal, 2014; Lee, 2016), make EBV-specific immune responses suitable as interesting surrogate markers for the immune function of the host (Karrer and Nadal, 2014).

EBV and accordingly the immunological reactions to the virus can be used as indicator of the current immune status (Gleeson et al., 2002; Pottgiesser et al., 2012). Thus, EBV and accordingly the immunological reactions to the virus could help to identify athletes with an increased susceptibility to infections (Bakker et al., 2007; Hoffmann et al., 2010). Despite immunological changes, obvious clinical symptoms, and further potential impairments of performance, do not necessarily occur. Lower EBV-specific IgG titers were detected in winter sports athletes compared to controls suggesting a weaker immune function in competitive athletes with lower control of EBV (Hoffmann et al., 2010), in addition, observed slightly elevated EBV-specific IgG titers over the competition season were interpreted as a reaction to increased EBV activity accompanying stress-induced diminished T-cell function (Hoffmann et al., 2010). However, similar results from other studies were missed (He et al., 2013). Furthermore, there has been an ongoing controversy whether EBV infections among elite athletes occur at a higher incidence than in the general population (Pottgiesser et al., 2006). Thus, the clinical relevance of EBV infections remains unclear, especially in competitive sports, because a clear relationship between training, EBV-specific parameters, clinical symptoms and performance could not be demonstrated consistently.

The challenge is to objectify individual resilience in order to control future burdens and thus to be able to counteract overloading at all levels. With a proven relationship, the training could be adjusted to prevent clinical complaints. First of all, it is necessary to identify a dependent cascade between physical, psychological, and environmental stress factors, their effects on immunological parameters and their association to performance and clinical symptoms. So far, only isolated aspects have been examined with different results (Fricker et al., 1999, 2005; Gleeson et al., 2000; Konig et al., 2000; Reid et al., 2004; Pyne et al., 2005; Walsh et al., 2011a,b; Hellard et al., 2015). Furthermore, past studies were mostly undertaken on heterogeneous collectives with a small number of cases and selection of inconsistent outcomes (Cox et al., 2008; Alonso et al., 2010; Mountjoy et al., 2010; Dvorak et al., 2011; Schwellnus et al., 2012; Theron et al., 2013).

To date, the relationship between stress parameters, immune status and clinical outcomes, and the characterization of each aspect, have not been sufficiently addressed, especially in young elite athletes. Due to this lack, a prospective study was initiated, in which selected parameters in detail and their relationships to each other were examined. The study involved young elite athletes who were monitored longitudinal and included a clinical assessment for known causes of impaired health and performance, the determination of immune reactions and a standardized collection of illness parameters. The following hypotheses should be investigated: (1) Due to the high training loads, already at a young age, the athletes show a higher susceptibility to infections and prevalence of URTI, and indicate an increased sense of stress compared to controls; (2) EBV-specific immune responses can be used as a potential biomarker of the overall immune status due to the high EBV seroprevalence in adolescence; (3) Differences in EBV-specific IgG titers will be detected in the young athlete collective compared to controls; (4) High training loads lead to an impaired stress sense and increase the incidence of clinical complaints; (5) Training load and stress affect the immune system; (6) The prevalence of upper respiratory tract infections (URTI) is associated with EBV serostatus and EBV-specific IgG titers.

Comprehensive data were collected to characterize the collective of young elite athletes. For the present analysis the following parameters were selected: training hours per week (training load), self-reported stress level, EBV serostatus and EBV-specific IgG titers (immune status), upper respiratory tract infection symptoms and susceptibility to infections (clinical outcome).

## Materials and methods

### Study design

**Figure 1:**
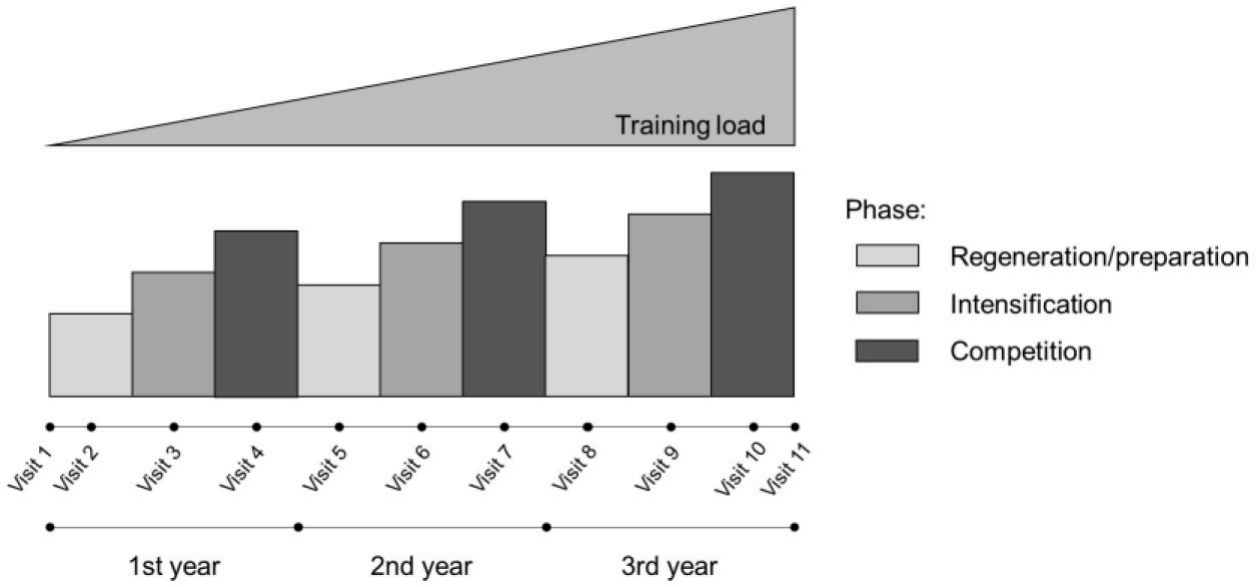
Timeline for the prospective study.

A controlled, prospective, longitudinal study was conducted between 2010 and 2014 with several regenerations, training and competitive seasons. Each year, the athletes were examined three times to determine the effects of certain stress factors (e.g., training load), plus their dynamics, on selected outcome measures (e.g., clinical, immunological, psychological end points). The study design included up to 13 visits per athlete. The timeline for the prospective surveillance study is shown in Figure 1. Comprehensive parameters for characterization of each athlete were determined once a year at one of three sports medicine centers (*Munich, Leipzig, Dresden*) to detect underlying conditions including case history, physical examination, anthropometry, clinical chemistry, ECG (electrocardiogram), echocardiography, and stress test. Additional baseline data and contained questionnaires that assessed e.g., training history, training loads, the WURSS-21 (21-item *Wisconsin Upper Respiratory Symptom Survey*), and health-related demands (self-reported health/stress sense, fatigue) were collected. As part of each visit, EBV serostatus and EBV-specific IgG titers were determined longitudinally.

### Participants

The athletes were recruited from different sport disciplines. Participation in the examinations (Munich, Leipzig or Dresden) was required. A total of 274 national class adolescent athletes (national junior top-level or comparable training level, age: 13.8 ± 1.5 years [yrs], male [m]: *n* = 175, female [f]: *n* = 99) from ten different sports (cross-country skiing [*n* = 43], cycling [*n* = 64], figure skating [*n* = 6], gymnastics [*n* = 4], high diving [*n* = 6], soccer [*n* = 72], speed skating [*n* = 11], swimming [*n* = 30], tennis [*n* = 7], volleyball [*n* = 31]) were enrolled in the study. The sports were categorized into three groups depending on the dynamic load (*low*: gymnastics, high diving; *medium*: figure skating, volleyball; *high*: speed skating, cycling, swimming, cross-country skiing, soccer, tennis; Mitchell and Haskell, 2005). The athletes were located in 18 training groups at seven locations. Prior to commencement of the investigations each athlete underwent a comprehensive clinical examination and was checked to assess inclusion (at V1 age ≤18 years; competing successfully at international or national level competitions for at least 2 years; belonging to one of the 18 training groups to ensure systematic training; future perspective of the athlete; written informed consent from parents and athletes) and exclusion criteria (chronic pathology or use of drugs that affected immune function; long-lasting injury or illness at V1). Athletes were prospectively followed for 2.2 ± 1.1 yrs. The maximum time between initial examination (V1) and the last visit (mean age: 16.1 ± 1.9 yrs) was 4 years. In addition, 285 control subjects (students, who did not perform competitive sports, age: 14.5 ± 1.9 yrs, m: *n* = 122, f: *n* = 163) were recruited. Athletes were tested up to ten times while controls had a maximum of two tests. In both groups, not all subjects could be tested at each time points resulting in some missing values. In total, 2.600 study days (athletes: 2.259, controls: 341) were collected. Depending on the analysis, data with inadequate detail in the recording were excluded. All athletes and controls were fully informed about the rationale for the study and of all procedures to be undertaken. Before baseline visit (V1) participants and their parents signed a written informed consent form. The study was approved by the medical research ethics committee (TU München) and it conforms to the principles outlined in the Declaration of Helsinki.

### Outcome and end point definitions

#### EBV serology

Blood samples were collected at each visit and peripheral blood was taken by puncture of the antecubital vein. EBV-specific IgG and IgM antibodies were measured by ELISA (Enzygnost®, Siemens Healthcare Diagnostics GmbH, Germany) according to the manufacturer's instructions. The assay detects antibodies directed against early EBV antigens (EA), viral capsid EBV antigens (VCA), and EBV nuclear antigen 1 (EBNA-1) in equal proportions. EBV-specific IgG and IgM antibodies were reported in units per milliliter (U/ml). The lower limit of detection was 25 U/ml. *Recom*Line® EBV IgG immunoblot assay (Mikrogen GmbH, Germany) detects antibodies against various EBV antigens (EBNA-1 [p72]), VCA [p18 and p23]), immediate-early antigen (BZLF-1), and EA [p138 and p54]). EBV-specific antibodies present in the cohort sample bound to these recombinant antigens and were detected by secondary antibodies directed against human IgG and coupled with horseradish peroxidase. Band signals were evaluated and stages of EBV infection (EBV serostatus) were differentiated according to the manufacturer's instructions in the following categories: EBV-seronegative (EBV–), EBV-seropositive (EBV+), suspicion of EBV reactivation (sRA), and suspected EBV infection (sNI).

#### Upper respiratory tract infection (URTI)

The WURSS-21 (21-item *Wisconsin Upper Respiratory Symptom Survey*) was used to compute symptoms of URTI (Barrett et al., 2005). This questionnaire is a responsive, reliable and valid instrument including 21 items (10 items: symptoms, 9 items: functional impairments, 1 item: global severity and global change over the last 24 h). All the items are responded to using a Likert scale of severity, ranging from 0 to 7. Symptoms not experienced were recorded as 0. An overall score (WTS) was calculated by adding the severity scores from the items 2–20 with high severity scores indicating high symptom load. An URTI episode was deemed present when total symptom score was greater seven, representing either one severe symptom or impairment or seven mild symptoms/impairments presented simultaneously. The occurrence of URTI symptoms were recorded at each visit every third day in an observation period of 2 weeks (5 times per visit). For representing the whole time span, all available scores were averaged.

#### Self-reported stress level, susceptibility to infection, training load

Each subject was asked to complete a questionnaire prior each examination among (1) stress level (%), (2) susceptibility to infection, and (3) training load (Th/w).

(1) To estimate the individual level of stress a visual analog scale (VAS) was used, with a range from 0 to 100 percent. A higher score indicated greater stress sense (0: “no stress,” 100: “highest stress level”). (2) In addition, athletes were asked if they felt sick more often (compared to the past/to others). The question could be answered with “*yes”*, “*no”*, “*I don't know.”* If the question was answered with “*yes,”* there was a subjective tendency to recurrent infections (susceptibility to infection). (3) For every visit, training loads were recorded after completing questionnaire and interview. In addition, the average number of training hours per week (Th/w) of the last 4 weeks was reported.

#### Statistical analyses

The data were compiled using Microsoft Excel® and evaluated using the SPSS® software (version 23.0; SPSS Lead Technologies Inc, Chicago, IL). Frequency distributions of all continuous variables were examined to detect outlying values, and the Kolmogorov-Smirnov test was used to check the normal distribution of variables. All results, assuming normal distribution, were presented as mean ± standard deviation (SD). Differences between groups were analyzed using an independent samples *t*-test. To determine the differences in the group analysis (e.g., WURSS-21), ANOVA was used. The chi-square test was performed to verify possible differences between nominal scaled variables. Significance was accepted at the *P* < 0.05 level. Depending on the analysis, data were stratified by sex and age, were presented by percentiles (using 10th and 90th percentile), and variables were categorized in ordinal gradation.

## Results

### Characterization (training load, stress level, susceptibility to infection, URTI) and comparison of the collectives (athletes vs. controls)

#### Training load

During the study period, the athletes (age: 15.1 ± 1.9 yrs) trained, on average, 14.9 ± 5.6 h weekly (>80% specific training for each sports discipline). Increasing training loads were significantly associated with a higher age (<10 h: 13.6 ± 1.8 yrs vs. ≥10 h: 15.4 ± 1.7 yrs, *p* < 0.001). A 16-year-old female figure skater achieved the maximum of 31.5 h. 22.3% of all, in particular 40.2% of the female athletes (m: 11.6%), completed at least 20 h of training per week, mostly belonging to gymnastics (25.9 ± 1.9 h), high diving (23.5 ± 4.6 h), or figure skating (22.8 ± 4.9 h). Remarkable, athletes of these three kind of sports were significantly younger compared to the others (13.7 ± 1.8 yrs vs. 15.2 ± 1.8 yrs, *p* = 0.002). Female volleyball players offered, despite younger age (f: 14.9 ± 1.5 yrs vs. m: 16.1 ± 1.0 yrs, *p* = 0.008), higher training loads compared to the male team (f: 20.4 h vs. m: 11.7 h, *p* < 0.001).

#### Stress level

Taking all athletes' data sets into account, a mean stress level of 45.3 ± 18.0% (MIN 0%, MAX 88.0%) was reported. Unlike the male participants, female athletes felt more stressed (f: 50.0 ± 15.7% vs. m: 42.5 ± 18.6%, *p* = 0.001). Similar results were found in the control group (f: 50.8 ± 25.9 % vs. m: 41.4 ± 23.9%, *p* = 0.005), who had in comparison to the athletes no lower stress levels (47.0 ± 25.5%, *p* = 0.387), in both sexes (f: *p* = 0.789, m: *p* = 0.668). The control subjects indicated a maximum stress level of 90.0%.

#### Susceptibility to infection

**Figure 2:**
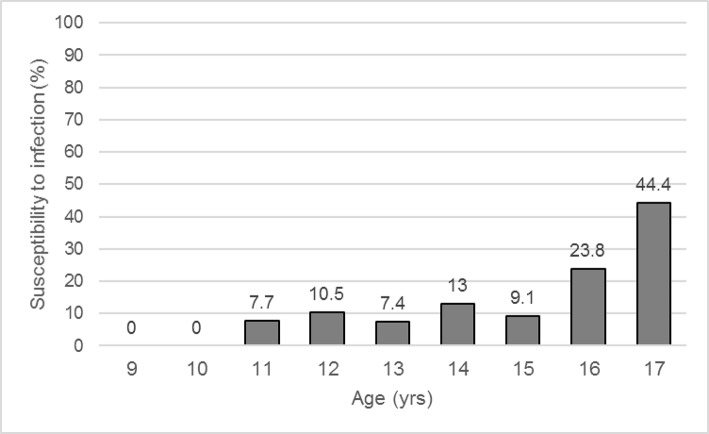
Prevalence of susceptibility to infection in athletes depending on age. Values are expressed as means.

At baseline visit 12.0% of competitive athletes (A) reported a subjective susceptibility to infection with no significant difference compared to the control (C) group (A: 12.0% vs. C: 8.5%, *p* = 0.153), in female (A: 18.2% vs. C: 12.3%, *p* = 0.188) as well as in male subjects (A: 8.6% vs. C: 3.3%, *p* = 0.064). Female athletes and female controls reported more often recurrent infections than males (A: f: 18.2% vs. m: 8.6%, *p* = 0.019; C: f: 12.3% vs. m: 3.3%, *p* = 0.006). Exemplary, no male volleyball player was anamnestic susceptible to infections, compared to 33.3% of the female players. While 11-year-old athletes had a prevalence of 7.7% for recurrent infections, this increased to 13.0% at the age of 14, and to 44% among 17-year-old participants (Figure 2). Athletes with recurrent infections (I+) were older compared to clinically unremarkable (I−) subjects (I−: 13.7 ± 1.5 yrs vs. I+: 14.4 ± 1.6 yrs, *p* = 0.016). During the total study process, while an observation period of 2.2 ± 1.1 yrs, 32.8% of all athletes reported recurrent illnesses, again with a significantly higher occurrence in the female group (f: 51.5% vs. m: 22.3%, *p* < 0.001).

#### URTI

The mean total WURSS-21 score (WTS) was 6.98 ± 10.25, with a highest value of 62.40. Gender differences did not occur (f: 7.28 ± 10.39 vs. m: 6.67 ± 10.12, *p* = 0.430). A total score greater seven (WTS > 7 at least in one of five surveys within one visit), and thus the presence of an URTI, was achieved in 30.7% of cases (f: 30%, m: 31.5%). Among these, the gender proportion was similar (f: 48.4%, m: 51.6 %, *p* = 0.674). No age dependencies could be shown, neither of WTS (*p* = 0.829), nor in terms of URTI prevalence (*p* = 0.957).

### EBV serostatus

At V1, 60.3% of the athletes (mean age: 13.8 ± 1.5 yrs) were EBV-seropositive, including 6.1% of the athletes with a serological suspected EBV reactivation (sRA), and 0.6% with a primary EBV infection (sNI). The proportion of EBV-seropositive subjects did not differ significantly between male and female athletes (m: 58.6% vs. f: 63.3%, *p* = 0.452). Male subjects were significantly younger (m: 13.6 ± 1.5 yrs vs. f: 14.0 ± 1.6 yrs, *p* = 0.041), but no significant differences regarding the percentage of EBV-seropositive female and male athletes were detected. Furthermore, EBV-seropositive (EBV+) athletes showed a similar age compared to EBV-seronegative (EBV–) subjects (EBV+: 13.8 ± 1.6 yrs vs. EBV–: 13.6 ± 1.4 yrs, *p* = 0.351), both in male (*p* = 0.166) and female participants (*p* = 0.695). 56.6% of the controls (mean age: 14.5 ± 1.9 yrs) were EBV-seropositive, resulting in no significant differences of the EBV serostatus compared to the athletes at baseline visit (*p* = 0.376). In line with the athlete group, no significant gender difference was observed within the control group (EBV+: m: 53.7% vs. f: 58.8%, *p* = 0.339). Age-depending analyzes between the athlete and control group revealed comparable numbers of EBV-seropositive subjects (e.g., age 13 yrs: A (*n* = 54): 42.6% vs. C (*n* = 36): 44.4%, *p* = 0.862; age 14 yrs: A (*n* = 77): 67.5 % vs. C (*n* = 64): 62.5%, *p* = 0.532; age 15 yrs: A (*n* = 54): 63% vs. C (*n* = 52): 61.5%, *p* = 0.880).

### EBV-specific antibody levels

**Figure 3:**
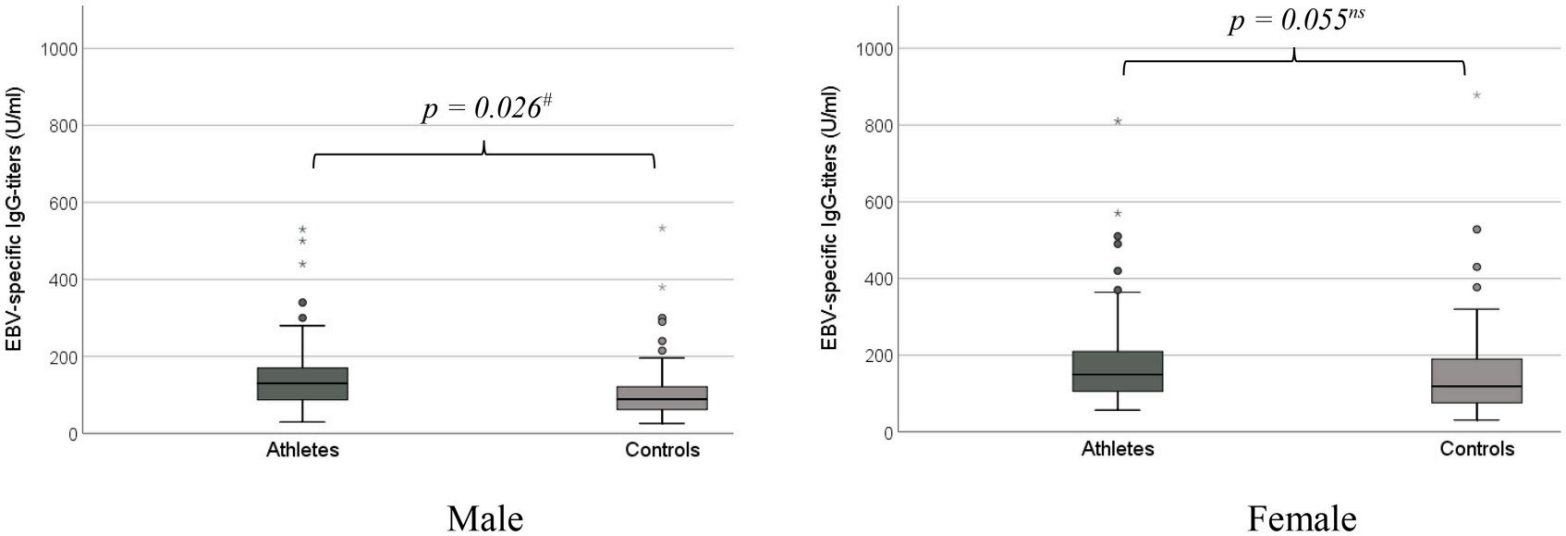
EBV-specific IgG-titers (U/ml) in comparison between athletes and controls. **(A)** male subjects. **(B)** female subjects. ^#^*p* < 0.05. ^*^Extreme value (defined as a value that is smaller (or larger) than 3 box-lengths).

In 145 athletes (m: *n* = 91, f: *n* = 54; age: 13.8 ± 1.6 yrs) EBV-specific IgG-titers (mean 166 ± 115 U/ml; range 30–810 U/ml) were detectable at baseline visit (V1). Female athletes showed significantly higher EBV-specific IgG titers compared to male athletes (f: 197 ± 145 U/ml vs. m: 147 ± 89 U/ml, *p* = 0.012), an age dependency was not proven (*p* = 0.365). Endurance athletes (high dynamic kind of sports) had the lowest EBV-specific IgG titers, compared to athletes of low and medium dynamic kind of sports (high: 153 ± 104 U/ml vs. medium: 225 ± 148 U/ml vs. low: 189 ± 138 U/ml, *p* = 0.025). The mean detectable EBV-specific IgG titers of the control group were 137 ± 112 U/ml (range: 26–878 U/ml), resulting in significant lower levels of EBV-specific IgG titers compared to the athletes at V1 (*p* = 0.030). This difference was confirmed in sub-analyzes of the male subjects (p = 0.026), but not in females (*p* = 0.055) (Figure 3). Similar to the athlete group, female control subjects showed higher EBV-specific IgG titers (C: m: 113 ± 90 U/ml vs. f: 152 ± 122 U/ml, *p* = 0.038), without any apparent age dependency (*p* = 0.839). The highest detectable EBV-specific IgG titer was measured in the collectives of the control subjects (C: 878 U/ml vs. A: 810 U/ml).

### It was investigated whether the subjective stress level, an increased susceptibility to infections, and the occurrence of URTI are related to the extent of training loads

#### Training load~stress level

With increasing training loads, defined as hours per week (Th/w), according to ordinal categorization (6 groups), a higher stress level was observed (<5 h: 42.2 ± 26.1%, 5–≤9.9 h: 41.8 ± 25.7%, 10 –≤14.9 h: 47.2 ± 24.3%, 15 –≤19.9 h: 52.9 ± 21.9%, 20–≤24.9 h: 54.4 ± 23.6%, ≥25 h: 61.4 ± 17.9%, *p* < 0.001), both in female (*p* = 0.007) and male athletes (*p* = 0.029). The 90th percentile of training load was 20 h per week, the 10th percentile was 6 h per week. Significant differences in stress sensation were identified between the three groups (≤6 h: 40%, >6– <20 h: 48.3%, ≥20 h: 55.6%, *p* < 0.001). This counted for both sexes, female (*p* = 0.007) and male athletes (*p* = 0.029).

#### Training load~susceptibility to infection

Based on the ordinal categorization, athletes with less than 5 h training load per week (Th/w) showed the highest prevalence of susceptibility to infections (20%). Overall, no significant differences between the groups were observed (<5 h: 20%, 5–≤9.9 h: 11.4%, 10–≤14.9 h: 11.1%, 15–≤19.9 h: 13.4%, 20–≤24.9 h: 12.8%, ≥25 h: 11.5%, *p* = 0.436), in both sexes (f: *p* = 0.113, m: *p* = 0.843). Athletes with a training load of at least 20 h per week (90th percentile), reported not more often recurrent infections compared to athletes with less strains (≤6 h: 13%, >6–<20 h: 12.4%, ≥20 h: 12.6%, *p* = 0.982).

#### Training load~URTI

Analyzes of WURSS-21 data revealed no ordinal relationships between training loads and WTS (<5 h: 8.74 ± 13.42, 5–≤9.9 h: 6.60 ± 9.16, 10–≤14.9 h: 7.36 ± 11.52, 15–≤19.9 h: 4.92 ± 7.21, 20–≤24.9 h: 6.21 ± 9.33, ≥25 h: 13.74 ± 16.81, *p* = 0.016), or rather URTI prevalence (<5 h: 30.8%, 5–≤9.9 h: 29.9%, 10–≤14.9 h: 30.8%, 15–≤19.9 h: 22.4%, 20–≤24.9 h: 31.3%, ≥25 h: 46.7%, *p* = 0.323). But, an obvious higher score (WTS) and URTI prevalence were seen at trainings loads of at least 25 Th/w. Taking into account the categorization according to percentiles, significant associations were also missing (URTI: ≤6 h: 25.8%, >6– <20 h: 28.2%, ≥20 h: 34.2%, *p* = 0.479), both, in female (*p* = 0.702) and male group (*p* = 0.146).

### In order to show a possible influence of training and stress on the immune system, training hours per week and subjective stress levels were compared with EBV-specific parameters

#### Training load~EBV serology

Training loads (Th/w) had no relation to EBV-serostatus (EBV–: 12.3 ± 5.3 h vs. EBV+: 13.0 ± 5.7 h, *p* = 0.057). After ordinal categorization of the training load (six groups), the relating mean EBV-specific IgG-titers were determined. No significant correlations between training hours per week and the extent of EBV-specific IgG-titers was observed (<5 h: 111 ± 75 U/ml, 5–≤9.9 h: 138 ± 99 U/ml, 10–≤14.9 h: 146 ± 110 U/ml, 15–≤19.9 h: 149 ± 113 U/ml, 20–≤24.9 h: 148 ± 113 U/ml, ≥25 h: 127 ± 45 U/ml, *p* = 0.364). Sub-analyzes of different training loads in female (*p* = 0.791), and male athletes (*p* = 0.380) revealed no gender-specific significant differences. Also after comparing the EBV-specific IgG-titers between the percentile groups, no significant differences were recognizable (≤6 h: 144 ± 104 U/ml, >6– <20 h: 147 ± 109 U/ml, ≥20 h: 117 ± 80 U/ml, *p* = 0.087). However, subgroup analyzes illustrated obvious lower EBV-specific IgG titers for athletes who trained at least 20 h per week (≥20 h: 117 ± 80 U/ml vs. >6– <20 h: 147 ± 109 U/ml, *p* = 0.028; ≥20 h; 117 ± 80 U/ml vs. ≤6 h: 144 ± 104 U/ml, *p* = 0.072).

#### Stress level~EBV serology

EBV-seropositive athletes reported significantly higher stress levels compared to EBV-seronegative subjects (EBV–: 44.8 ± 24.4% vs. EBV+: 49.1 ± 25.0%, *p* = 0.004). This difference was confirmed in female athletes (*p* = 0.002), but not in the male collective (*p* = 0.393). After subdividing the subjects in different age groups, no significant differences regarding the stress level of EBV-seronegative and EBV-seropositive athletes were observed. Exemplary, in the subgroup of 18-year-old subjects, EBV-seronegative athletes showed stress levels of 54.0 ± 5.5%, in contrast to 46.1 ± 22.9% of EBV-seropositive athletes (*p* = 0.450). After further serological differentiation, athletes with suspected EBV reactivation (sRA) or primary EBV infection (sNI) offered the highest levels of stress sense (EBV–: 44.8 ± 24.4%, EBV+: 48.5 ± 24.9%, sRA: 58.8 ± 24.0%, sNI: 57.1 ± 25.8%, *p* = 0.004). Based on ordinal categorization of the stress levels (five groups), relative EBV-specific IgG-titer were assessed and compared with each other. Differences between the groups were not found, neither in the overall cohort (≤10%: 129 ± 83 U/ml, >10–≤30%: 128 ± 83 U/ml, >30–≤50%: 143 ± 106 U/ml, >50–≤70%: 150 ± 117 U/ml, >70%: 142 ± 127 U/ml, *p* = 0.279), nor in the female (*p* = 0.303) or male group (*p* = 0.186). Analyzes based on the percentiles yielded comparable results (≤10%: 129 ± 83 U/ml, >10–80%: 141 ± 105 U/ml, ≥80%: 143 ± 133 U/ml, *p* < 0.601).

### Finally, the relations between EBV-specific parameters and clinical outcomes were investigated

#### EBV serology~susceptibility to infection

12.4% of EBV-seronegative and 11.8% of EBV-seropositive athletes reported recurrent infections. The difference was not significant, both in female (*p* = 0.178) and male sub-collectives (*p* = 0.575). Exemplary, 25.0% of EBV-seronegative athletes above 18 years declared susceptibility to infections, in contrast to 16.7% of EBV-seropositive subjects (*p* = 0.681). Closer examination of clinically conspicuous athletes showed a prevalence rate of 63.7% compared to a prevalence rate of 64.9% in healthy athletes (*p* = 0.769). These results were confirmed regardless of age- and gender-based sub-analyzes. Athletes with recurrent infections had no significant different levels of EBV-specific IgG-titers compared to clinically healthy subjects (I–: 139 ± 102 U/ml vs. I+: 160 ± 120 U/ml, *p* = 0.078), in both genders (f: I–: 151 ± 121 U/ml vs. I+: 179 ± 135 U/ml, *p* = 0.118; m: I–: 129 ± 84 U/ml vs. I+: 120 ± 63 U/ml, *p* = 0.580). The highest rates of susceptibility to infections were found in athletes within the 90th percentile group of EBV-specific IgG-titers, but with no significance (≤51 U/ml: 9.6% vs. >51– <268 U/ml: 10.3% vs. >268 U/ml: 15.3%, *p* = 0.415).

#### EBV serology~URTI

**Figure 4:**
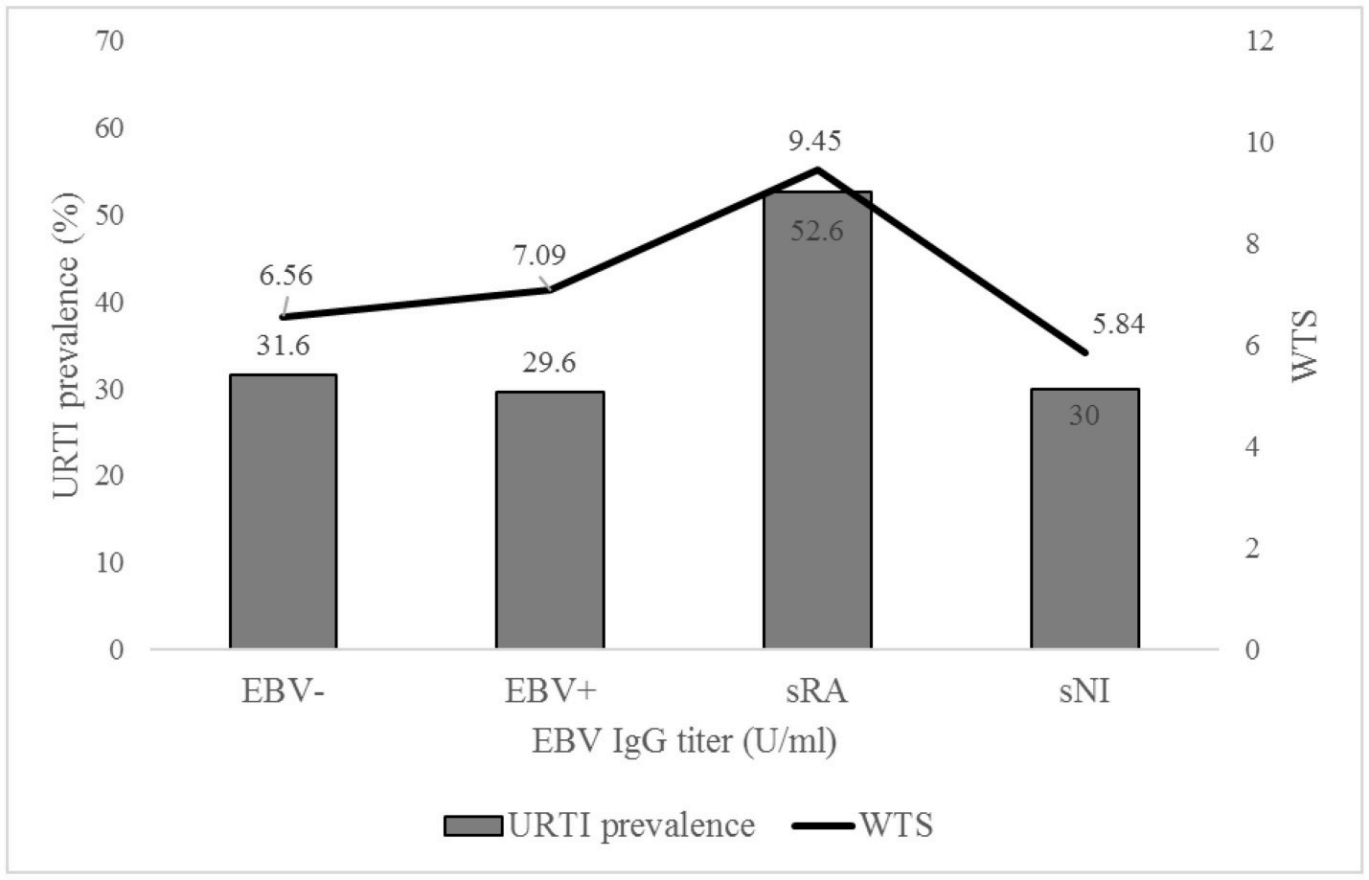
URTI prevalence and WURSS-21 total score (WTS) depending on EBV serostatus (EBV–: EBV-seronegative [*n* = 253], EBV+: EBV-seropositive [*n* = 392], sRA: suspected reactivation [*n* = 19], sNI: suspected new infection [*n* = 5]). Values are expressed as means.

**Figure 5:**
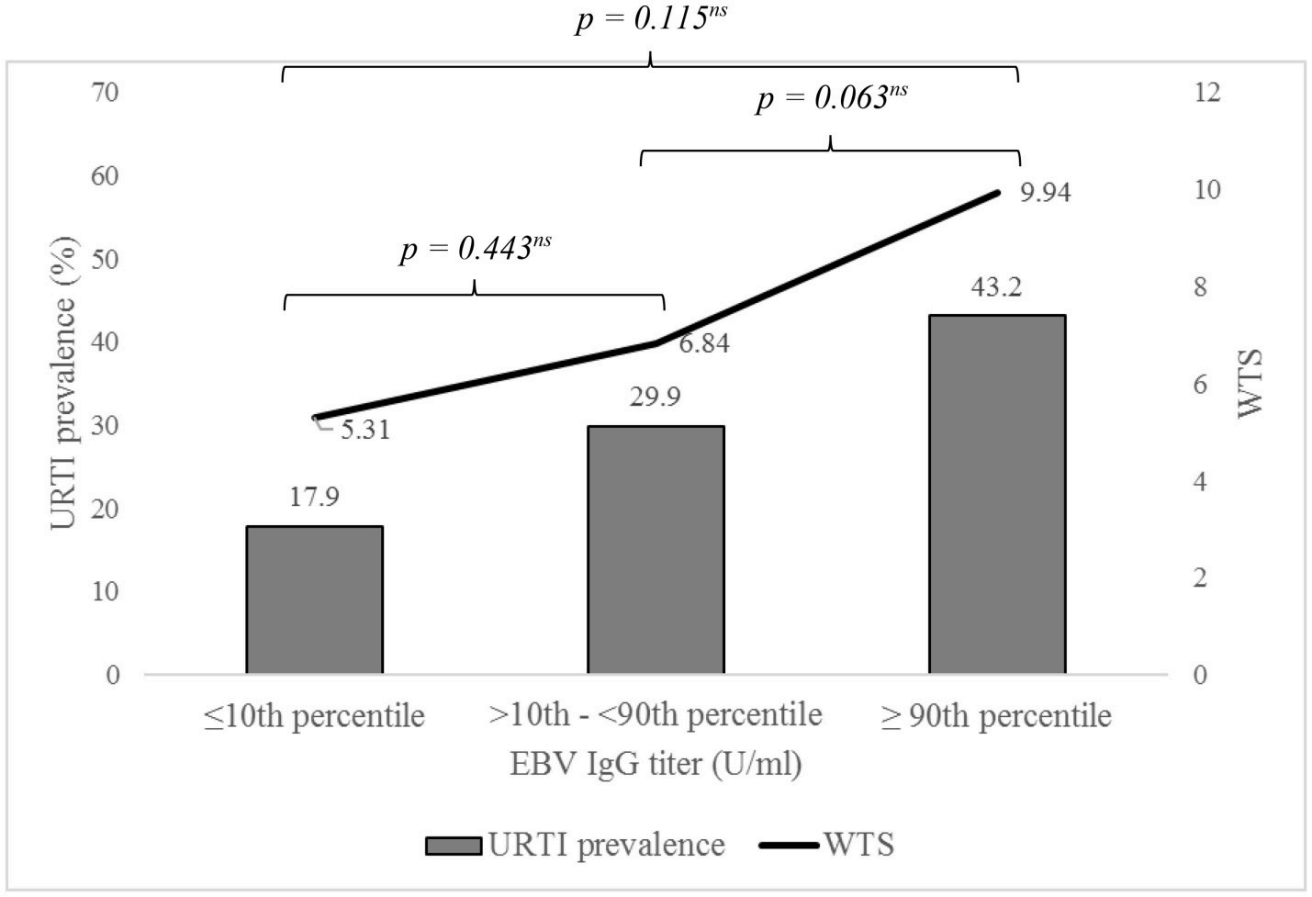
URTI prevalence and WURSS-21 total score (WTS) depending on EBV-specific IgG titers categorized in different percentiles (≤10th percentile: ≤51 U/ml, >10th-<90th: >51-<268 U/ml, ≥90th: ≥268 U/ml). Values are expressed as means.

There was no relation between EBV serostatus and extent of WTS (EBV–: 6.59 ± 9.40 vs. EBV+: 7.14 ± 10.48, *p* = 0.470), in both genders (f: *p* = 0.128, m: *p* = 0.529). Also, the URTI prevalence (WTS >7) between the two groups did not differ (EBV–: 31.6% vs. EBV+: 30.2%, *p* = 0.486). This was confirmed in all age ranges (≤12 yrs: EBV–: 28.6% vs. EBV+: 31.8%, *p* = 0.791; 13–14 yrs: EBV–: 31.5% vs. EBV+: 28.8%, *p* = 0.661; 15–16 yrs: EBV–: 33.3% vs. EBV+: 31.7%, *p* = 0.773; 17–18 yrs: EBV–: 28.0% vs. EBV+: 26.3 %, *p* = 0.874; >18 yrs: EBV–: 100% vs. EBV+: 36.4%, *p* = 0.460). 62.8% of the athletes with reported URTI symptoms were EBV seropositive, 64.3% of athletes without an URTI (*p* = 0.696). Athletes with serological suspected EBV reactivation showed the highest WTS, but no significant difference in comparison to the other groups was found (EBV–: 6.56 ± 9.40, EBV+: 7.09 ± 10.57, sRA: 9.45 ± 8.70, sNI: 5.84 ± 8.07, *p* = 0.641; Figure 4). The same was shown for the URTI prevalence (EBV–: 31.6%, EBV+: 29.6%, sRA: 52.6%, sNI: 30.0%, *p* = 0.183). Subjects with conspicuous WTS (>7) did not differ in EBV-specific IgG-titers from the unremarkable participants (URTI–: 137 ± 101 U/ml vs. URTI+: 158 ± 114 U/ml, *p* = 0.082), neither in female (URTI–: 164 ± 122 U/ml vs. URTI+: 170 ± 127 U/ml, *p* = 0.393) nor in male subjects (URTI–: 121 ± 74 U/ml vs. URTI+: 143 ± 95 U/ml, *p* = 0.100). In further analyzes, the URTI prevalence was determined as a function of the percentiles. An ordinal increase in prevalence with higher EBV-specific IgG-titers was evident, but at no time with any significance (URTI: ≤51 U/ml: 17.9%, >51– <268 U/ml: 29.9%, ≥268 U/ml: 43.2%, *p* = 0.064l; Figure 5).

In addition, presentation of the results was made in tabular form (Tables 1–3).

**Table 1:** Clinical parameters of EBV-seronegative and -seropositive athletes.

	**EBV-seronegative**	**EBV-seropositive**	***P***
Age (yrs)	13.6 ± 1.4	13.8 ± 1.6	=0.351
Stress level (%)	44.8 ± 24.4	49.1 ± 25.0	=0.004^*^
Training load (Th/w)	12.3 ± 5.3	13.0 ± 5.7	=0.057
Recurrent Infections (%)	12.4	11.8	=0.769
WTS	6.59 ± 9.40	7.14 ± 10.48	=0.470
URTI prevalence (%)	31.6	30.2	=0.696

*WTS, WURSS-21 total score; URTI; Upper respiratory tract infection. Data are shown as mean ± SD and percentage values*.

* *p < 0.01*.

**Table 2:** Clinical and immune parameters dependent on training load (training hours per week).

**Training load (Th/w)**	**Stress level (%)**	**Recurrent infections (%)**	**WTS**	**URTI (%)**	**EBV-specific IgG (U/ml)**
<5	42.2 ± 26.1	20	8.74 ± 13.42	30.8	111 ± 75
5 –≤9.9	41.8 ± 25.7	11.4	6.60 ± 9.16	29.9	138 ± 99
10 –≤14.9	47.2 ± 24.3	11.1	7.36 ± 11.52	30.8	146 ± 110
15 –≤19.9	52.9 ± 21.9	13.4	4.92 ± 7.21	22.4	149 ± 113
20 –≤24.9	54.4 ± 23.6	12.8	6.21 ± 9.33	31.3	148 ± 113
≥25.0	61.4 ± 17.9	11.5	13.74 ± 16.81	46.7	127 ± 45
	<0.001^**^	=0.436	=0.016^#^	=0.323	=0.364

*WTS, WURSS-21 total score; URTI, Upper respiratory tract infection. Data are shown as mean ± SD and percentage values*.

# *p < 0.05*,

** *p < 0.001*.

**Table 3:** Clinical parameters and training loads dependent on EBV-specific IgG titers categorized in different percentiles (≤10th percentile: ≤51 U/ml, >10th-<90th: >51-<268 U/ml, ≥90th: ≥268 U/ml).

	**EBV-specific IgG (U/ml)**			
	**≤10th percentile**	**>10th-<90th percentile**	**≥90th percentile**	***p***
Stress level (%)	52.8 ± 24.1	47.6 ± 25.1	52.0 ± 23.7	=0.093
Training load (Th/w)	12.2 ± 5.5	13.1 ± 5.8	13.2 ± 5.2	=0.429
Recurrent Infections (%)	9.6	10.3	15.3	=0.415
WTS	5.31 ± 11.60	6.84 ± 9.94	9.94 ± 12.25	=0.117
URTI prevalence (%)	17.9	29.9	43.2	=0.064

*WTS, WURSS-21 total score; URTI, Upper respiratory tract infection. Data are shown as mean ± SD and percentage values*.

## Discussion

The major aim of the study was to determine the interaction between training load, stress level, immune status, and clinical endpoints. In advance, an independent evaluation of each parameter, with the relationships to each other, were necessary to characterize the cohort of young elite athletes. Therefore, a large prospective study was initiated involving young elite athletes who were monitored longitudinally in terms of clinical relevant parameters.

On the way becoming a top athlete, many barriers are to overcome. Success needs a high degree of health, because less lack of practice for exercising the physiological demands is necessary (Armstrong and Mc Manus, 2011; Hastmann-Walsh and Caine, 2015). In particular, adolescence needs an increased attention (Sabato et al., 2016). First, because of the high relative increase in training in this timeframe, on the other hand due to the consideration of additional factors influencing individual development, such as school, parental conflicts, puberty, or other physical and psychological strains (Cohn, 1990; Scanlan et al., 1991; Puffer and McShane, 1992; Gould et al., 1993; Puente-Diaz and Anshel, 2005). For the athlete, a safe passage through this phase with presumed health and continuous training must be ensured (Mårtensson et al., 2014). Therefore, knowledge of necessary conditions, at all levels (e.g., clinical, physical, psychological), are essential (Sabato et al., 2016). Although junior sports have become more and more the focus of science and public in recent years, large prospective studies characterizing young athletes in this regard are missing (Armstrong and Mc Manus, 2011). The knowledge of all influencing factors, including their weighting, makes it possible to regulate them in order to avoid negative consequences.

High-performance sport is associated with physical strains. To ensure an optimal development, systematic increases in training loads, from adolescence to adulthood, are necessary to avoid overtraining or negative health outcomes (Sabato et al., 2016). In certain kind of sports, high levels of training are mandatory even at a young age (Armstrong and Mc Manus, 2011). On the one hand, e.g., gymnastics need physical requirements, which are limited in adulthood, on the other hand, e.g., tennis players have to learn difficult skills already in the early age. So it is not uncommon from an age of 12–13 years to train 15–20 h per week (Armstrong and Mc Manus, 2011). Our study examined 274 junior athletes from ten different sports. Already at a mean age of 15 years, the average training load was nearly 15 h per week, with a maximum of over 30 h. One in five athletes (22.3%) trained at least 20 h a week. There were marked differences in training loads between the athletes, depending on age, gender, kind of sports, and localization. The results illustrated that young athletes offer high training loads, comparable to adults. In addition to the training, also school lessons are to complete resulting in over 60 h overall strain per week. Despite the same sport, age, and gender, the strains can vary clearly and, therefore, have to be evaluated individually. Since young athletes show similar training loads as top-level adults, an adequate infrastructure (e.g., medical network) should already be available as early as possible.

Training can alter the immune system, by inducing a temporary immunosuppression (Reid et al., 2004; Fricker et al., 2005; Helenius et al., 2005; Tiollier et al., 2005; Cox et al., 2008), finally developing clinical complaints (Fricker et al., 2000; Konig et al., 2000). Competitive sport is associated with an increased risk for illness and injury (Armstrong and Mc Manus, 2011; Hastmann-Walsh and Caine, 2015). Here, upper respiratory tract infections belong to the most common reasons for impaired health and a diminished ability for regeneration (Schwellnus et al., 2016a). In certain circumstances, recurrent infections can result in frequent interruptions, lack of performance, and possibly retirement from competitive sports, furthermore, influencing long-term everyday life (Fricker, 1997; Alonso et al., 2010; Engebretsen et al., 2010, 2013; Mountjoy et al., 2010; Schwellnus et al., 2011, 2016a; Soligard et al., 2014). Based on the temporary immunosuppression, we assumed, that athletes show a higher susceptibility to infections, an increased prevalence of URTI, due to the high training loads already at a young age, and indicate an impaired sense of stress compared to controls (hypothesis 1). To prove these assumptions and to characterize each young elite athlete concerning this matter, following parameters were systematically examined and evaluated: (a) subjective stress level, (b) susceptibility to infections, (c) upper respiratory tract infection symptoms.

(a) Stress can negatively affect health (Novas et al., 2002; Putlur et al., 2004; Main et al., 2010). Therefore, the general aim is to minimize stress factors. In competitive sports, high training loads are inevitable, which can increase the subjective sense of stress and, thus, adversely affect the athletes' health. The cascade between extent of training, stress level, and health, respectively performance, is still unclear. Remarkable, our analyzes showed no increased sense of stress among the athletes compared to the control group, despite higher training loads and therefore increased strains. This illustrates the individual and multi-factorial etiology of stress. The counteracting positive effects of exercise on stress level can be assumed (e.g., increased vagotonus, structured everyday life, social environment, recognition, mental stability). In accordance to the literature, female subjects, in both cohorts, showed higher stress levels. In further analyzes, athletes with increased subjective stress levels have to been selected. After determining them, triggering risk factors can be avoided. Further investigations are needed to identify such triggering stress factors for developing appropriate interventions (Schwellnus et al., 2016a).

(b) Studies found an increased rate of recurrent infections in athletes due to a diminished transient immune competence after high-intensity or rather extensive training loads (Peters and Bateman, 1983; Fricker et al., 1999; Gleeson et al., 2000; Spence et al., 2007). In contrast, moderate exercise reduces the incidence of infections compared to physical inactivity (Matthews et al., 2002). This relation between extent of exercise and infection prevalence is described as J-shaped curve. To each visit, athletes and controls were asked how frequent they felt sick. At baseline visit a susceptibility to infections affirmed twelve percent of the athletes. There was no significant difference to the controls, maybe justified by the young age, fewer years of training, and, therefore, less chronic effects on the immune system. However, a gender difference was detected with a higher prevalence of subjective recurrent infections in female subjects, both in athletes and controls. Recent studies have shown fewer episodes of infections in successful top-ranking athletes, mentioned as S-curve relationship (Mårtensson et al., 2014; Hellard et al., 2015). In the future, the question must be clarified whether a certain adaptation of the immune system is responsible for this, the results are based on a selection mechanism, or the outcome is compatible with a better lifestyle (Schwellnus et al., 2016a). It must be noted, that our results are based on self-reported data. The clinical relevance of such subjective susceptibility to infection remains to be investigated. An increased physical focus of the athlete compared to the general population is conceivable. However, in our analyzes no higher prevalence of recurrent infections between both collectives was found.

(c) In addition to the subjective susceptibility to infections, the WURSS-21 was used to compute symptoms of URTI as an objective and validated clinical endpoint (Barrett et al., 2005). After calculation of the WURSS-21 total scores (WTS), no differences depending on age and sex were observed. Overall, a maximum value of 133 could have been achieved. In contrast, during analyzed observation a mean WURSS-21 score of under seven was found, leading to the assumption of an investigated collective with less clinical complaints. Fewer chronical effects of long-lasting and intensive training loads on immune system are feasible reasons for the results. On the other hand, there might be a different perception of exercise in adolescence (e.g., less pressure to succeed, “playful” component), with the used questionnaire symptoms and functional impairments were recorded. Furthermore, the objectivization of influencing the performance in practice remains questionable and needs more investigation.

The persistence of EBV in the organism after primary infection, the lifetime prevalence of more than 90%, the simple measurement of infection stage after an antigen-antibody-reaction, the practicable detection of a systemically increased EBV activity in whole blood or saliva, and the known associations to clinical parameters, although with inconsistent results, make EBV and accordingly the immunological reactions to the virus as interesting markers of the overall immune function of the host (Gleeson et al., 2002; Pottgiesser et al., 2012; Karrer and Nadal, 2014). In our own preliminary work, we examined EBV-specific serological parameters in adult elite athletes (Pottgiesser et al., 2006, 2012; Hoffmann et al., 2010). To clarify the use of EBV-specific immune responses as a potential marker of the immune status (hypothesis 2), we investigated the EBV seroprevalence in our recruited adolescent subjects. The examined athletes showed, at a mean age of approximately 14 years, an EBV seroprevalence of already 60%. Serostatus differences did not depend on sex and age. Particularly, in controls a similar prevalence rate was found (56.6%). The missing difference confirmed previous data (Pottgiesser et al., 2006; Hoffmann et al., 2010). So, the incidence of primary infection, and thus seroconversion, does not depend on the level of physical training, and argue against an increased EBV seroprevalence in athletes (Hoffmann et al., 2010). The elicited EBV prevalence corresponds with the literature (Pottgiesser et al., 2012). Because of the already high rate of seroprevalence in adolescence, EBV-specific parameters can be used as potential immune markers for further analyzes.

Illness, impaired performance, and fatigue are often associated with EBV infection (Balfour et al., 2015). High-intensity or rather extensive training loads transiently diminish the immune competence (Peters and Bateman, 1983; Fricker et al., 1999; Gleeson et al., 2000; Spence et al., 2007). Encouraging this assumption, lower EBV-specific IgG tiers were detected in studies with competitive athletes compared to controls (Hoffmann et al., 2010). In this regard, the authors suggested a weaker immune function in competitive athletes. Therefore, we assumed lower EBV-specific IgG titers in young elite athletes compared to controls (hypothesis 3). However, we measured higher antibody titers in the samples of young athletes. These data suppose a necessary chronic influence of training loads on immune system for obvious alterations, e.g., lower antibody titers. Otherwise, the elevated EBV-specific IgG titers are a result of a reaction to increased EBV activity accompanying stress-induced diminished T-cell function (Hoffmann et al., 2010). So, a reduced T-cell function allows higher EBV loads, and in turn stimulating IgG production. To prove the effects, an additional determination of viral loads (EBV DNA) and EBV-specific T-cell responses, in a longitudinal study design, and the consideration of different seasonal time points are necessary. These was done in the present study. However, we refer to future publications regarding these results. Sub-analyzes proved the lowest IgG titers in endurance athletes in comparison to subjects of sports with a medium or low dynamic load. Possibly, the missing IgG titer differences to controls are due the high activity of the students in this age range. In summary, our results showed no differences in EBV serostatus and no lower EBV-specific IgG titers in athletes compared to controls in adolescent age.

Consistent data on the relationship between physical, psychological, and environmental stress factors, their effects on immunological parameters and their association to performance and clinical symptoms are missing. After analyzing parameters (training load, subjective stress level, EBV seroprevalence, EBV-specific IgG titers, susceptibility to infections, and upper respiratory tract infection symptoms) for themselves, the associations to each other have been addressed.

At first, we inspected if high training loads lead to an impaired stress sense and increase the incidence of clinical complaints (hypothesis 4). With increasing training loads, athletes felt more stressed, both in female and male athletes. The results were expected and can be justified with the higher time effort of training sessions. Contrariwise, controls showed no lower subjective stress levels compared to athletes despite more leisure time. Athletes offered an average training load of nearly 15 h per week corresponding with a mean stress level under 50 percent. In contrast, athletes with at least 25 h per week reported a level above 60%. So, a cut-off value of hours per week is to be assumed. The missing difference to the controls illustrated moreover the necessary consideration of further triggering factors, which cause conspicuous stress sense. Consequently, the positive and negative aspects of competitive sports should always be taken into account. In addition to the presence of high strains, the individual handling of these should also be assessed. For further sub-analyzes athletes with high stress levels but low training loads will be selected. In addition, the influences of the extent of training load on infection-related parameters were examined. Neither the occurrence of subjective susceptibility to infection nor the presence of relevant clinical symptoms (URTI) were affected by the amount of exercise. Athletes with training loads of at least 25 h per week showed an obvious higher rate of an URTI (46.7%). However, an ordinal gradation of occurrence did not depend on training load. It is important to mention, that athletes with training loads under 5 h per week were mostly ill, resulting in high prevalences of clinical parameters in this group. In summary, no direct relationship between training loads and clinical outcomes were found. In further analyzes, ill and injured athletes should be excluded.

Published studies documented an altered immune system depending on strains (Dennis et al., 2005; Dun et al., 2005; Fleisig et al., 2011). We assumed an influence of training loads and stress sense on the immune system, hence, on EBV-specific parameters (hypothesis 5). In the examined young athlete collective, significant associations between training loads or rather stress levels and EBV-specific serological parameters, as potential immune markers, were absent. In our preliminary work we found lower EBV-specific IgG titers in competitive athletes compared to controls (Hoffmann et al., 2010). This could reflect weaker immune function because of high training loads. In the present study, EBV-specific IgG levels from young athletes do not correlate to training loads and subjective stress levels. Unique, athletes with a suspected EBV reactivation showed significantly higher stress levels compared to the others. For an adequate evaluation, further multivariate subgroup analyzes with the addition of other relevant parameters (e.g., EBV DNA, CRP, leucocytes) will be achieved.

Clinical relevant symptoms, such as recurrent infections and fatigue, are often associated with EBV (Balfour et al., 2015). So, studies with competitive athletes showed an association between viral load and the frequency of respiratory infections (Gleeson et al., 2002). Based on that, we assumed a relationship between the prevalence of upper respiratory tract infections (URTI) and EBV-serostatus or rather EBV-specific IgG titers. In the conducted analyzes, neither the prevalence of upper respiratory tract infections (WTS > 7) nor the occurrence of susceptibility to infections are associated with EBV-serostatus or EBV-specific IgG titer levels. High IgG titers tended to show conspicuous clinical complaints, but at no time with a significance. Particularly, athletes with serological suspected EBV reactivation (sRA) presented the highest prevalence for URTI, even if there was no significant difference between the groups.

In summary, young elite athletes offered high training loads comparable to adults. Athletes showed no increased sense of stress, no higher prevalence of recurrent infections, and no different EBV-specific serological parameters compared to controls. With increasing training loads athletes felt more stressed, but significant associations to EBV-specific serological parameters were absent. Also no direct relationships between training loads and clinical outcomes with EBV-specific immune responses were found.

Due to the high strain variability, each athlete has to be evaluated individually, taking into account all influencing factors, and needs an adequate medical infrastructure to avoid negative long-term outcomes. After their objectification, inappropriate loads should be avoided by early detection and following interventions. In synopsis of the results, the occurrence of clinical symptoms cannot be established by training loads alone. Furthermore, EBV serostatus and the level of EBV-specific antibodies do not allow risk stratification for infections. Further investigations are needed, in particular subgroup-analyzes of athletes at risk and the addition, respectively the consideration, of other parameters (e.g., viral load, EBV-specific T-cell responses, performance data, CRP, leucocytes, psychological parameters). One strength of our study was that numerous parameters, in an interdisciplinary approach, were determined to characterize the collective of adolescent athletes compared to controls. In contrast to previous studies, upper respiratory tract infection symptoms were interrogated with a validated questionnaire (WURSS-21). In further analyzes other blood parameters for infection detection will be used. Otherwise, results of susceptibility to infections, training loads, and stress levels based on self-reported assessments. To our knowledge, we present the first prospective, controlled study of elite young athletes to examine the relationship between training load, stress parameters, immune status and clinical outcomes on such a large-scale cohort.

## Author contributions

KB and BW conceived of the presented idea. KB and BW developed the theory and performed the computations. KB and BW verified the analytical methods. All authors discussed the results and contributed to the final manuscript.

### Conflict of interest statement

The authors declare that the research was conducted in the absence of any commercial or financial relationships that could be construed as a potential conflict of interest. The handling Editor declared a shared affiliation, though no other collaboration, with one of the authors KB and BW.
